# Mycobacterium tuberculosis infection as a zoonotic disease: transmission between humans and elephants.

**DOI:** 10.3201/eid0402.980217

**Published:** 1998

**Authors:** K Michalak, C Austin, S Diesel, M J Bacon, P Zimmerman, J N Maslow

**Affiliations:** McHenry County Department of Health, Woodstock, Illinois 60098, USA.

## Abstract

Between 1994 and 1996, three elephants from an exotic animal farm in Illinois died of pulmonary disease due to Mycobacterium tuberculosis. In October 1996, a fourth living elephant was culture-positive for M. tuberculosis. Twenty-two handlers at the farm were screened for tuberculosis (TB); eleven had positive reactions to intradermal injection with purified protein derivative. One had smear-negative, culture-positive active TB. DNA fingerprint comparison by IS6110 and TBN12 typing showed that the isolates from the four elephants and the handler with active TB were the same strain. This investigation indicates transmission of M. tuberculosis between humans and elephants.


### *Mycobacterium tuberculosis* Infection as a Zoonotic Disease: Transmission between Humans and Elephants

**Kathleen Michalak,* Connie Austin,† Sandy Diesel,* J. Maichle Bacon,* Phil Zimmerman,‡ and Joel N. Maslow§**
*McHenry County Department of Health, Woodstock, Illinois, USA; †Illinois Department of Public Health, Springfield, Illinois, USA; ‡University of Illinois, College of Medicine at Rockford, Rockford, Illinois, USA; and §Boston University School of Medicine and the VA Medical Center, Boston, Massachusetts, USA

Between 1994 and 1996, three elephants from an exotic animal farm in Illinois died of pulmonary disease due to *Mycobacterium tuberculosis.* In October 1996, a fourth living elephant was culture-positive for *M. tuberculosis.* Twenty-two handlers at the farm were screened for tuberculosis (TB); eleven had positive reactions to intradermal injection with purified protein derivative. One had smear-negative, culture-positive active TB. DNA fingerprint comparison by IS*6110* and TBN12 typing showed that the isolates from the four elephants and the handler with active TB were the same strain. This investigation indicates transmission of *M. tuberculosis* between humans and elephants.

*Mycobacterium tuberculosis* and *M. bovis*, related organisms of the *M. tuberculosis* complex, infect a wide variety of mammalian species. *M. bovis* is pathogenic for many animal species, especially bovidae, cervidae, and occasionally carnivores. Human disease with *M. bovis* is well described and historically was a common cause of tuberculosis (TB) transmitted by infected dairy products. As a result of milk pasteurization and TB eradication programs in most industrialized countries, zoonotic transmission of *M. bovis* through domestic livestock is now rare. In contrast, a similar eradication program has not been conducted for wild or exotic animals, which therefore remain an uncommon source for *M. bovis* exposure. Zoonotic transmission of *M. bovis* has been reported from seals, rhinoceros, and elk (1-4).

*M. tuberculosis*, the most common species to cause TB, classically causes disease in humans. Animal infection with *M. tuberculosis*, while uncommon, has been described among species (e.g., birds, elephants, and other mammals) with prolonged and close contact with humans (5-10). Transmission of *M. tuberculosis* between animals and humans has not been reported. This paper describes *M. tuberculosis* transmission from elephants to humans.

Address for correspondence: Kathleen Michalak, McHenry County Department of Health, 2200 N. Seminary Avenue, Woodstock, IL 60098, USA; fax: 815-338-7661.

### The Outbreak

In March 1996, five elephants from an exotic animal farm in Illinois were in California as part of a circus act. One elephant (with chronic, unexplained weight loss since October 1995) died under anesthesia on August 3, 1996, during a diagnostic dental work-up. Necropsy showed widespread consolidation of lung tissue with caseous necrosis of the lungs and mediastinal lymph nodes. Short, fat, relatively scant numbers of acid-fast bacilli were observed in necropsy tissues. A presumptive diagnosis of *M. tuberculosis* was made. The remaining four elephants were recalled to the farm in Illinois. A second elephant died en route on August 6, 1996. Necropsy revealed copious respiratory and trunk exudates and caseous necrosis of the lung.

To determine the risk for and possibility of infection among the animal trainers and caretakers, an epidemiologic investigation was initiated. The remaining elephants in the herd and the elephant handlers and trainers who were still traveling were recalled to the farm and examined for evidence of *M. tuberculosis* infection. All elephants were empirically begun on antituberculous therapy in early December 1996.

### Epidemiologic Investigation

The exotic animal farm was visited on numerous occasions to evaluate the type and degree of contact between elephants and employees. The farm, located in a rural area and surrounded by barbed wire and trees, originally housed 18 Asian and 2 African elephants. Thirteen elephants were tethered on a chain in one large barn, four were housed in a separate large room (two in a common stall), and a baby elephant was in a third room with 5-6 tigers. A separate barn housed approximately 80 tigers.

### TB Screening of Employees

The animal handlers (trainers and caretakers) who had direct contact with the elephants were administered purified protein derivative (PPD) skin tests. Initial screening was performed in August 1996, with subsequent screenings in December 1996 and March, June, and September of 1997. Testing was performed by the McHenry County Department of Health, except in two handlers who had subsequent skin tests performed elsewhere. As part of the screening process, handlers were questioned about their risk factors for TB, including previous bacillus Calmette-Guérin (BCG) vaccination.

Handlers were tested by the two-step method using 5 tuberculin units of PPD (0.1 ml) by intradermal injection in the flexor surface of the forearm. A positive result was defined as an induration of >5 mm. Handlers with positive skin tests were evaluated by a TB health-care worker and had chest radiographs taken. Sputum samples from any handler with a chest radiograph consistent with TB were submitted to the Illinois Department of Public Health Laboratory. Samples were examined by direct microscopy for acid-fast organisms, stained with fluorochrome, and processed for culture by standard methods.

### Examination of Isolates

The human isolate and the four elephant isolates were sent to the National Tuberculosis Genotyping and Surveillance Network at the Michigan Community Public Health Agency for restriction fragment length polymorphism (RFLP) analysis. Southern blots of Pvu II restricted whole chromosomal DNA, resolved in 1% agarose gels, were probed with a DNA fragment corresponding to the right side of IS*6110* and detected by chemiluminescence (11). The number and size of the hybridizing fragments for each isolate were compared in the same gel. Isolates with identical RFLP patterns or with ≤ 2 band differences were considered to represent the same strain. Additionally, Pvu II digested DNA was similarly typed after probing with the repetitive element TBN12.

### Epidemiologic Findings

Elephant handlers worked in very close proximity with the elephants around the clock, whereas tiger handlers had little direct contact with the elephants. Most of the elephant handlers lived on the farm in a separate section of the barn; four lived in trailers on the grounds. The handlers' living quarters had a separate ventilation system from the elephants'; however, the doors between the two quarters were open for unknown periods. Handlers indicated that they held social events in a building connected to the elephant barn.

Necropsies of elephants were performed on the farm and were attended by a number of elephant and tiger handlers (including the handler with the active case). The necropsy of the elephant that died in 1994, also performed on the farm, showed caseous necrosis of the lungs and pleural exudates whose culture yielded *M. tuberculosis*.

In addition to the three elephants that died of *M. tuberculosis* infection, a fourth living elephant was also infected with the mycobacterium; this infection was diagnosed in late December 1996 from a trunk culture obtained in October 1996. Subsequent cultures from this and the other animals have been negative for mycobacteria. Another elephant from this farm died of *M. tuberculosis* infection in 1981 (5), but contact between this elephant and the present herd or any of the handlers could not be established.

Twenty-two handlers at the exotic animal farm had moderate to frequent contact with the infected animals; 12 were elephant handlers and 10 were tiger handlers. Initial PPD testing was performed for 14 handlers in August 1996, 2 in October 1996, and 5 in December 1996. One who was PPD-positive in November 1995 reported receiving BCG more than 10 years before.

Eleven (50%) of 22 handlers were found PPD-

positive as part of this investigation. Eight of the 11 had positive PPD skin test results upon initial testing, with a median induration of 12 mm (range, 10 to 19 mm). Four of the eight were elephant handlers and four were tiger handlers. The skin test reaction of three handlers converted from negative to positive with a median induration of 12 mm (range, 8 to 15 mm). The three PPD converters were initially tested in August 1996; one was positive upon retesting in January 1997, and two tested positive in April 1997 (Table).

Eight of the 11 handlers reported that they had negative skin tests in the past and had not received BCG. The other three reported some type of reaction from a previous skin test in the past but did not know the results. All three also reported receiving BCG more than 10 years before. Eight of the 10 handlers with negative PPD skin tests had at least one negative follow-up test at 3 months; two left the farm and did not receive follow-up testing.

The attack rates were approximately equal for the elephant and tiger handlers. Of the 12 elephant handlers tested, 6 (50%) were PPD-positive with two conversions documented in April 1997; of the 9 tiger handlers, 5 (56%) were PPD-positive, with one conversion documented in January 1997. Overall, a very high rate (52%) of handlers tested positive.

All 12 handlers with positive PPDs (including the one with the known positive PPD) received an evaluation and chest radiograph; one had irregular nodules and interstitial changes in the right apex without retraction of the lungs, consistent with active TB, but no cough, chest pain, fever, night sweats, weight loss, or fatigue.

Three sputum samples were smear-negative for acid-fast bacilli, although one yielded *M. tuberculosis* upon culture. Isoniazid (INH), rifampin, pyrazinamide, and ethambutol treatment was initiated in September 1996, and after 2 months, was reduced to INH and rifampin when the isolate showed no resistance to antituberculous medications. Subsequent chest radiographs revealed improvement or clearing of the initial lesions. Nine of the remaining 11 PPD-positive handlers were prescribed INH prophylaxis; two declined because of the risk for adverse reactions.

Table. TB PPD[a] skin test results of animal handlers, Aug 1996–Sep 1997

| | Positive | Negative |
|---|---|---|
| Previously positive | 1 | |
| Elephant handlers | 4 | 6 |
| Tiger handlers | 4 | 4 |
| Elephant handlers (converted) | 2 | |
| Tiger handlers (converted) | 1 | |
| Total | 12 | 10 |

[a]Tuberculin purified protein derivative.

### Molecular Analysis of Elephant and Human Isolates

The sputum isolate from the handler with active TB was compared with the isolates from the three animals that died and the living elephant whose infection was diagnosed during the investigation. The isolates had identical IS*6110* RFLP pattern, differing by ≤ 2 bands (Figure 1). Additionally, all isolates had the identical TBN12 RFLP pattern, except the isolate from the elephant that died in August 1996, which demonstrated a shift of one band (Figure 2).

### Conclusions

Infection with *M. tuberculosis* or *M. bovis* has not been reported in nondomesticated Asian or African elephants. *M. tuberculosis* infection in domesticated elephants was first reported in 1875 by Garrod and has been recognized in the ancient Ayurvedic literature (10); humans have been considered the source of infection. A trainer with cavitary TB was suspected as the source of infection (8) for one Asian elephant that died of *M. tuberculosis*, although subsequent analysis showed the animal and human isolates to be of two different phage types.

This report describes the first case of zoonotic *M. tuberculosis* transmission. The epidemiologic investigation strongly suggests *M. tuberculosis* transmission between humans and elephants, as evidenced by DNA fingerprinting. RFLP analysis comparing Southern blots of chromosomal DNA probed with IS*6110* and TBN12 indicated that four elephant isolates had identical patterns with the human isolate, differing by ≤ 2 bands. The addition or loss of a single band has been demonstrated in other outbreak settings, and the repetitive element that generates patterns has characteristics of a mobile genetic element (11).

Eleven (50%) of 22 employees screened were skin-test positive, with no difference between tiger and elephant handlers. This is a higher rate of positives than documented in animal handlers exposed to *M. bovis*-infected animals (3,4). Since the handlers had no accurate

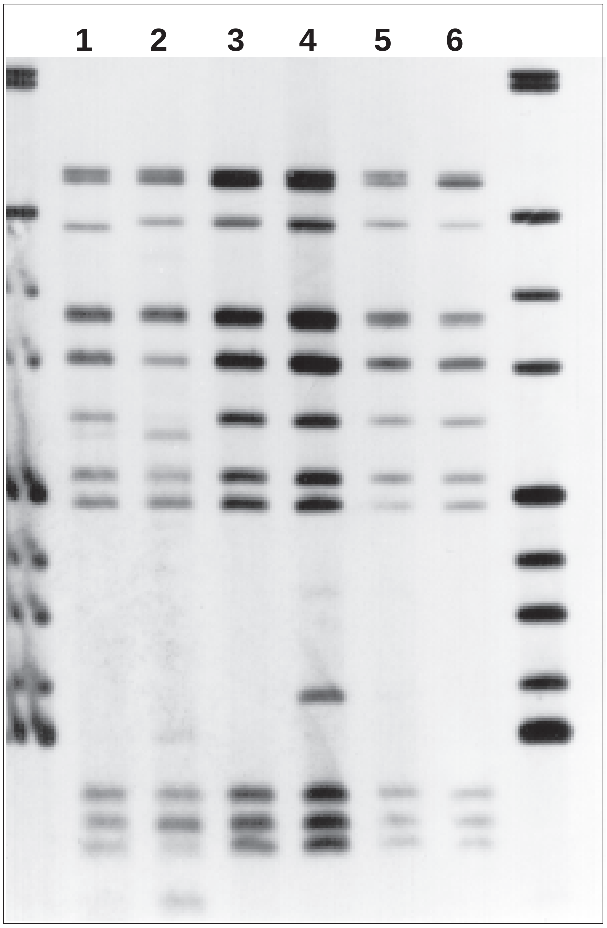

Figure 1. IS*6110* restriction fragment length polymorphism results. Lane1, elephant isolate (died August 6, 1996); Lane 2, elephant isolate (died 1994); Lane 3, living elephant trunk culture (October 1996); Lane 4, elephant lung tissue isolate (died August 3, 1996); Lane 5, elephant lymph node tissue isolate (died August 3, 1996); Lane 6, human sputum isolate (September 1996). Provided by State of Michigan Community Public Health Agency.

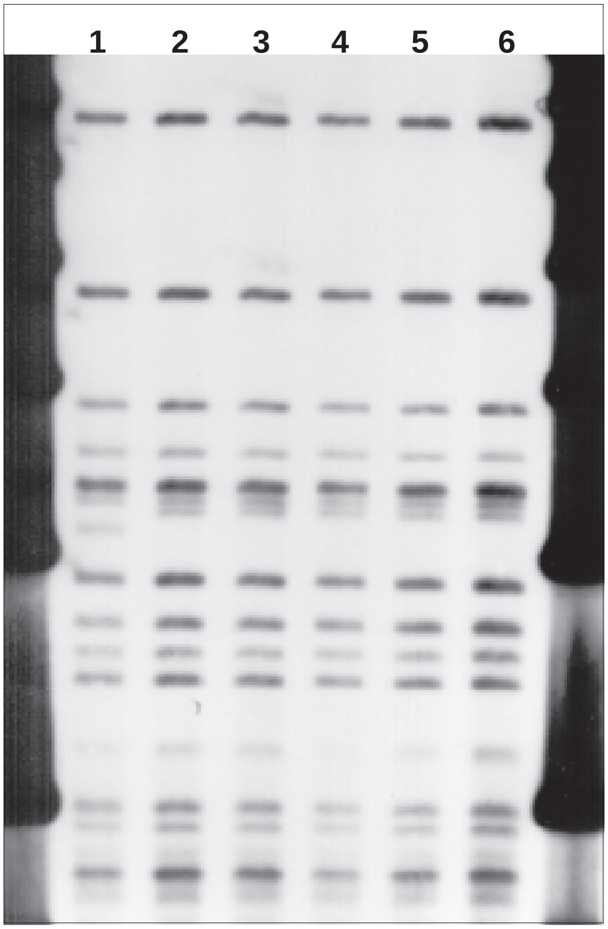

Figure 2. TBN12 restriction fragment length polymorphism results. Lane1, elephant isolate (died August 6, 1996); Lane 2, elephant isolate (died 1994); Lane 3, living elephant trunk culture (October 1996); Lane 4, elephant lung tissue isolate (died August 3, 1996); Lane 5, elephant lymph node tissue isolate (died August 3, 1996); Lane 6, human sputum isolate (September 1996). Provided by State of Michigan Community Public Health Agency.

history of tuberculin skin testing, it was not possible to determine when conversions took place. The original source of infection for both elephants and humans is unknown.

The possible mechanisms of transmission include close contact while handling and training elephants, cleaning the barn, participating in elephant necropsies, and living in close proximity to the elephant barn.

Human-to-human transmission of TB is unlikely because the only handler with active disease did not have cough. Of the three sputum samples initially collected, two were smear- and culture-negative; the third had low numbers of acid-fast bacilli manifested by a negative sputum smear, thus posing a low infectivity risk to others. In contrast, the three elephants that died had evidence of widespread pulmonary disease and, therefore, represented a greater risk for dissemination.

Three handlers converted from negative to positive during the course of the investigation; their relevant exposure is unknown. The source may have been one elephant found antemortem to be culture-positive for M. *tuberculosis*, although this animal did not return to the farm until November. Contact with this animal is unlikely for handlers whose PPD tests converted in December and unknown for the two handlers whose test results were positive in April (the latter two had not been retested since August).

TB is transmitted through close prolonged contact with a person (or animal) with active TB. The risk for TB transmission from an animal with a case of active TB is higher for daily handlers than for persons with only brief contact, e.g., members of the public viewing a performance or receiving elephant rides. In this outbreak, screening of all persons who had (or thought they had) contact with an elephant that died of *M. tuberculosis* identified three PPD-positive cases but no cases of active TB (8). Because the real risk for transmission to the general public was poorly understood, this case received considerable media attention as well as mention in the medical literature (7,12).

Veterinary practices should be initiated to reduce the risks for exposure to animals infected with *M. tuberculosis*. No data are available on TB incidence among domesticated elephants in the United States. An estimate can be derived from a retrospective study of 379 zoo elephants of which eight (2.3%) had *M. tuberculosis* infection (10).

Reliable diagnosis and prevention of TB in all domesticated and exhibited animals is ideal. Short of this, possible ways to prevent and decrease zoonotic spread of any mycobacterial infection (*M. tuberculosis* or *M. bovis*) include 1) regular skin testing of handlers or keepers; 2) a high index of suspicion of TB in elephants with unexplained weight loss, cough, or rhinorrhea; 3) public health measures of contact tracing and notification; and 4) active and effective treatment of infected personnel and animals (13).

### Acknowledgments

We thank Stephen Dietrich and Laura Mosher, North Central Area TB-RFLP Laboratory, Michigan Department of Public Health; George J. Dizikes, Chief of Molecular Diagnostics, Illinois Department of Public Health, Division of Laboratories; and Janet Payeur, National Veterinary Services Laboratory, U.S. Department of Agriculture, MB Section. This research was supported in part by the Centers for Disease Control and Prevention, National Tuberculosis Genotyping and Surveillance Network cooperative agreement.

Kathleen Michalak is the Director of Nursing for McHenry County Department of Health. She has worked to improve the use of community resources, and improved service to client, providers, and the community as a whole.

### References

1. Thoen CO. Tuberculosis (Revised 1995). Zoonosis updates. Journal of the American Veterinary Medical Association 1995;2:155-8.
2. Thompson PJ, Cousins DV, Gow BL, Collins DM, Willamson BH, Dagnia HT. Seals, seal trainers, and mycobacterial infection. American Review of Respiratory Diseases 1993;147:164-7.
3. Dalovisio JR, Stetter M, Mikota-Wells S. Rhinoceros' rhinorrhea: cause of an outbreak of infection due to airborne *Mycobacterium bovis* in zookeepers. Clin Infect Dis 1992;15:598-600.
4. Fanning A, Edwards S. *Mycobacterium bovis* infection in human beings in contact with elk (*Cervus elaphus*) in Alberta, Canada. Lancet 1991;338:1253-5.
5. Saunders G. Pulmonary *Mycobacterium tuberculosis* infection in a circus elephant. J Am Vet Med Assoc 1983;183:1311-2.
6. Pinto MRM, Jainudeen MR, Panabokke RG. Tuberculosis in a domesticated Asiatic *Elaphas maximus*. Vet Rec 1973;93:662-4.
7. Frankel D. Elephants pack their trunks and leave the circus. Lancet 1997;349:1675.
8. Greenberg AS, Jung RC, Gutter AK. Hazel Elephant is dead (of Tuberculosis). American Review of Respiratory Diseases 1981;124:341.
9. Jones WD, Good RC. Hazel elephant redux. American Review of Respiratory Diseases 1982;125:270.
10. Mikota S, Sargent EL, Ranglack GS. Medical management of the elephant. Tuberculosis and Tuberculin Testing 1994;33-9.
11. Small P, Moss A. Molecular epidemiology and the new tuberculosis. Infect Agents Dis 1993;2:132-8.
12. Furley CW. Tuberculosis in elephants. Lancet 1997;350:224.
13. Centers for Disease Control and Prevention. Guidelines for preventing the transmission of *Mycobacterium tuberculosis* in health-care facilities. MMWR Morb Mortal Wkly Rep 1994;43:63-4.
